# Mechanisms of utilisation of arabinoxylans by a porcine faecal inoculum: competition and co-operation

**DOI:** 10.1038/s41598-018-22818-4

**Published:** 2018-03-14

**Authors:** Guangli Feng, Bernadine M. Flanagan, Deirdre Mikkelsen, Barbara A. Williams, Wenwen Yu, Robert G. Gilbert, Michael J. Gidley

**Affiliations:** 1 ARC Centre of Excellence in Plant Cell Walls, Centre for Nutrition and Food Sciences, Queensland Alliance for Agriculture and Food Innovation, The University of Queensland, St Lucia, QLD 4072 Australia; 2 Centre for Nutrition and Food Sciences, Queensland Alliance for Agriculture and Food Innovation, The University of Queensland, St Lucia, QLD 4072 Australia; 3 Joint International Research Laboratory of Agriculture and Agri-Product Safety, College of Agriculture, Yangzhou University, Yangzhou, Jiangsu Province 225009 China

## Abstract

Recent studies show that a single or small number of intestinal microbes can completely degrade complex carbohydrates. This suggests a drive towards competitive utilisation of dietary complex carbohydrates resulting in limited microbial diversity, at odds with the health benefits associated with a diverse microbiome. This study investigates the enzymatic metabolism of wheat and rye arabinoxylans (AX) using *in vitro* fermentation, with a porcine faecal inoculum. Through studying the activity of AX-degrading enzymes and the structural changes of residual AX during fermentation, we show that the AX-degrading enzymes are mainly cell-associated, which enables the microbes to utilise the AX competitively. However, potential for cross-feeding is also demonstrated to occur by two distinct mechanisms: (1) release of AX after partial degradation by cell-associated enzymes, and (2) release of enzymes during biomass turnover, indicative of co-operative AX degradation. This study provides a model for the combined competitive-co-operative utilisation of complex dietary carbohydrates by gut microorganisms.

## Introduction

Digestion of dietary carbohydrates in humans and other monogastric animals is limited to simple sugars and starch^1^. Complex dietary carbohydrates, generally originating from plant cell walls, remain undigested in the stomach and small intestine, passing to the large intestine, where they are fermented by the resident microbiota^2^. This microbiota has evolved to utilise these dietary carbohydrates for their own nutrition. In return, short-chain fatty acids and other by-products of the microbiota are a source of energy and critical nutrients for the large intestinal epithelial cells^3^. In addition, recent research suggests that cardiovascular disease^4^, non-alcoholic fatty liver disease^5^, obesity^5,6^, type 2 diabetes^7^ and inflammatory bowel diseases^8^ are each associated with alterations in the compostion of gut microbiota, which is affected by the fermentation of dietary fibre^9,10^. An understanding of how to manipulate the function and microbial composition is expected to lead to health improvements, either by disease prevention and/or treatment. This has inspired studies investigating the metabolism of complex dietary carbohydrates by the gut microbiota^1,9,11–14^. Using new technologies (including culture-independent genomics and metagenomics technologies), the diversity of the microbiota present, and the numerous carbohydrate-degrading enzymes, have started to be characterised^8^. With the discovery of polysaccharide utilisation loci (PULs), various polysaccharide metabolism-based studies have progressed significantly, and polysaccharide utilisation systems encoded by PULs of individual gut symbionts have been established^1,9,14^. However, unlike an individual microorganism, the gut microbiota is a dynamic ecosystem containing a diverse range of species interacting with each other, where microbial functions might differ greatly depending on the presence or absence of other community members^11^. Presently, understanding dietary fibre fermentation in this environment has focussed on co-culture experiments of selected microbial species, in the presence of dietary fibres^9,15,16^. However, considering the hundreds of species which constitute the gut microbiota^17^, and the long-term adaption of microbes in this competitive environment, it is essential that the entire microbiota be included to fully appreciate the mechanisms of dietary fibre fermentation. Knowing how the microbiota work as a community is one of the prerequisites to deploying strategies to manipulate this microbial population and hence maximise human health benefits.

Xylans are the second most abundant structural polysaccharide in plant cell walls after cellulose, and are particularly prominent in the cell walls of cereal grains^18^. Arabinoxylan (AX), one of the main classes of xylan^9^ and a prominent dietary polysaccharide in wheat and rye^19,20^, exhibits prebiotic effects such as: improved immunomodulatory properties^19^, restoration of a balanced bacterial composition^21^ and control of obesity and related metabolic disorders^22^. In the last few decades, colo-rectal cancer has increased in more proximal colonic regions, the site where soluble and rapidly digestible polysaccharides are more likely to be fermented^23^.

This paper describes the mechanisms by which the faecal microbiota ferment water-soluble wheat and rye AX *in vitro*, using a porcine faecal inoculum. Pigs are an ideal model for studies related to human digestive function and nutrition, sharing similar gastro-intestinal anatomical features and microbial populations^24,25^, and because they can be fed a controlled diet. In this study, the fermentation of wheat and rye AX was studied using a functional enzyme assay approach in order to (a) define the location within microbes of the three activities required to degrade AX to monomeric arabinose and xylose (*endo*-β−1,4-xylanase, β-D-xylosidase, α-L-arabinofuranosidase), (b) determine whether AX is fully digested once associated with the microbiota, using size exclusion chromatography of the soluble polymers, and (c) identify the mechanisms by which enzymes can become solubilised from microbes. The combination of these studies allows the proposal of a combined competitive-co-operative fermentation model for AX, as an example of how a complex dietary carbohydrate is broken down by a gut microbial population.

## Results and Discussion

### Soluble AX is rapidly fermented

Analysis by ^1^H NMR of wheat (WAX) and rye AX (RAX) reveals a β-1,4 linked xylan backbone that is substituted singly at C(O)3 or doubly at C(O)2 and C(O)3 with arabinofuranosyl moieties. WAX has a higher percentage of di-substitution, while RAX arabinose is mostly mono-substituted (Table 1).

**Table 1:** Structural properties of the WAX and RAX through ^1^H NMR analysis.

	A/X ratio	Mono-Xyl^a^	Di-Xyl^b^	Un-Xyl^c^	Purity (DM basis)	DM^d^
WAX	0.55	0.19	0.18	0.63	96%	92.89%
RAX	0.48	0.31	0.08	0.61	91%	91.90%

^a^Percentage of mono-substituted xylose units; ^b^percentage of di-substituted xylose units; ^c^percentage of un-substituted xylose units; ^d^dry matter.

**Figure 1:**
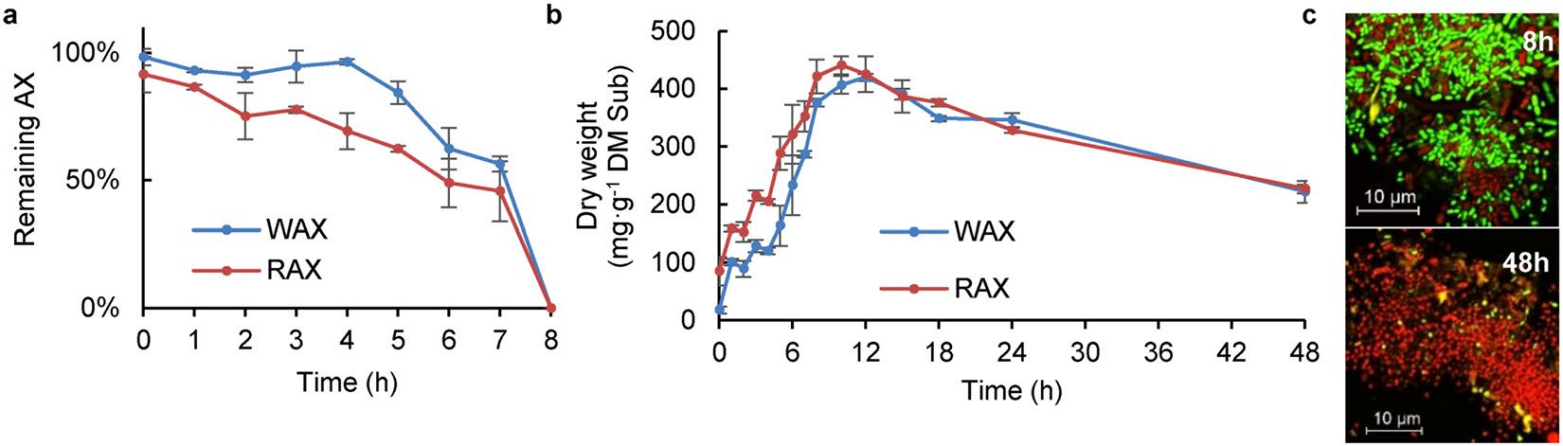
Fermentation of wheat and rye arabinoxylans with a porcine faecal inoculum. (**a**) Remaining AX in the culture medium; data are expressed as the percentages compared with the control sample containing the substrate and the medium but no inoculum (AX_Med). WAX = wheat arabinoxylan; RAX = rye arabinoxylan. (**b**) Dry weights of the microbial pellet during fermentation. (**c**) Images from confocal laser scanning microscopy of microbes post-fermentation for 8 and 48 h. Microbes were stained with the live/dead bacterial viability kit (BacLight, L7012), in which microbes with intact membranes are stained green with SYTO 9 while microbes with damaged membranes are stained red in the presence of propidium iodide.

During fermentation, residual AX in the culture medium decreased, with neither WAX nor RAX being detected after 8 h (Fig. 1a). The increased dry weight of microbes accompanying the depletion of AX (Fig. 1b) indicates active microbial growth fuelled by fermentation of AX. Microbial dry matter decreased 12 h later, presumably as a result of cell autolysis due to nutrient depletion after AX digestion. This was confirmed by confocal laser scanning microscopy (CLSM) images showing that after 8 h fermentation, microbes were mostly viable (stained green), while by 48 h, most of the cells had damaged membranes (stained red) (Fig. 1c).

From Fig. 1, it can be seen that the fermentative profiles of WAX and RAX were similar, though RAX was fermented faster in the first five hours, as indicated by its more rapid depletion from the medium (Fig. 1a), as well as increased microbial growth (Fig. 1b). This suggests that the faecal microbiota ferment the AX with fewer di-substitutions faster. However, the profiles of short chain fatty acid (SCFA) and NH_3_ production between WAX and RAX are similar (see Supplementary Fig. S1), indicating that fermentation products are not necessarily directly associated with fermentation rate.

### During active AX fermentation, enzymes are not secreted into the medium

**Figure 2:**
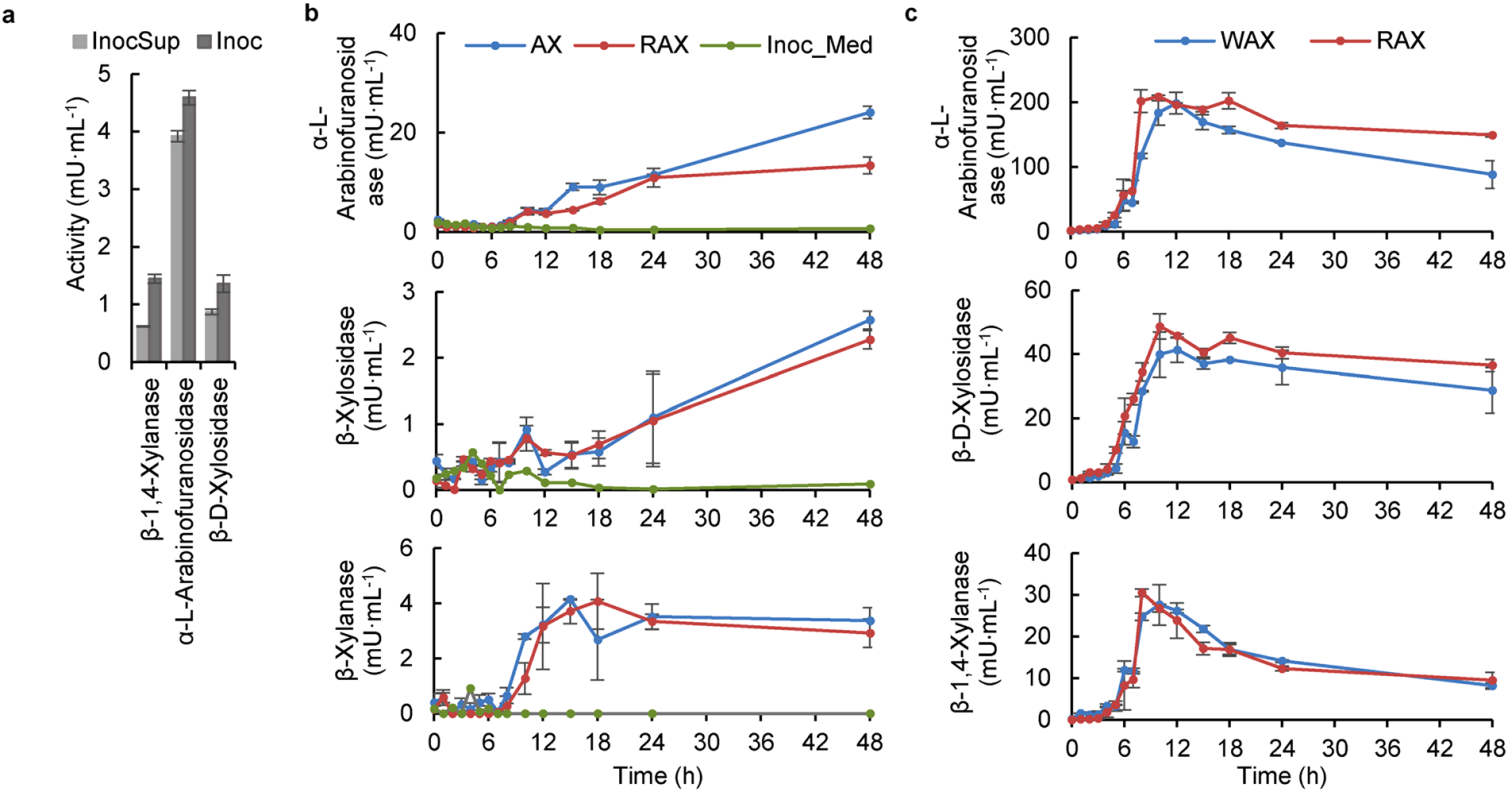
Enzyme activities. (**a**) Enzyme activities in the inoculum. InocSup = inoculum supernatant, obtained by centrifugation of the inoculum for 10 min at 5,000 g and 4 °C; Inoc = whole inoculum without centrifugation. (**b**) Enzyme activities in the culture medium during fermentation. WAX = wheat arabinoxylan; RAX = rye arabinoxylan. Inoc_Med was the control sample containing the inoculum and the medium but without the substrate. (**c**) Enzyme activities of the microbial pellets using whole-cell assays.

Although the pigs were on a controlled diet containing no AX for ten days prior to faeces collection for inoculum, activities of α-L-arabinofuranosidase (4.59 ± 0.12 mU/mL), β-1,4-xylanase (1.45 ± 0.08 mU/mL) and β-D-xylosidase (1.36 ± 0.15 mU/mL) were detected in the inoculum (Fig. 2a). Most of the enzyme activity (85%) was detected in the inoculum supernatant for α-L-arabinofuranosidase, whereas for the other two enzymes, activity in the supernatant was less dominant (64% for β-D-xylosidase and 43% for β-1,4-xylanase; Fig. 2a). Also, for the first 8 h of fermentation (the period of active fermentation, Fig. 1a), enzyme activities in the culture medium were comparable between bottles with and without AX (Fig. 2b), indicating that these enzymes were most likely from the inoculum supernatant. However, after degradation of AX was complete, enzyme activities increased, a potential result of enzyme release due to microbial autolysis. This is supported by CLSM micrographs (Fig. 1c), showing the autolysis of microbes at 48 h. It has been reported previously that enzymes might be released into the environment during biomass turnover^26–28^. Thus before fermentation, enzymes present in the inoculum, specifically in the supernatant, were likely from autolysed microbes that were capable of degrading AX.

### AX-degrading enzymes are microbial cell wall-associated

Whole cell assays indicated that the degrading enzymes are cell-associated, as the activity levels (Fig. 2c) were much (typically > 20×) higher than in solution, particularly during the active fermentation of AX. The enzyme activity profiles in microbial pellets showed a 3 h lag phase, followed by a rapid increase in enzyme activity, consistent with the rapid depletion of residual AX from 3 h onwards. The enzyme profiles for WAX and RAX fermentations were similar, except for the significantly higher (*P* < 0.0001) α-L-arabinofuranosidase activity detected during RAX fermentation at 8 h, consistent with the faster degradation of RAX. Similar results have been obtained using human faeces, showing better growth of microbes on RAX hydrolysate (higher in single arabinose substitution and lower in double substitution levels than WAX – Table 1) than AX oligosaccharide (AXOS) doubly substituted with arabinose^29^.

**Figure 3:**
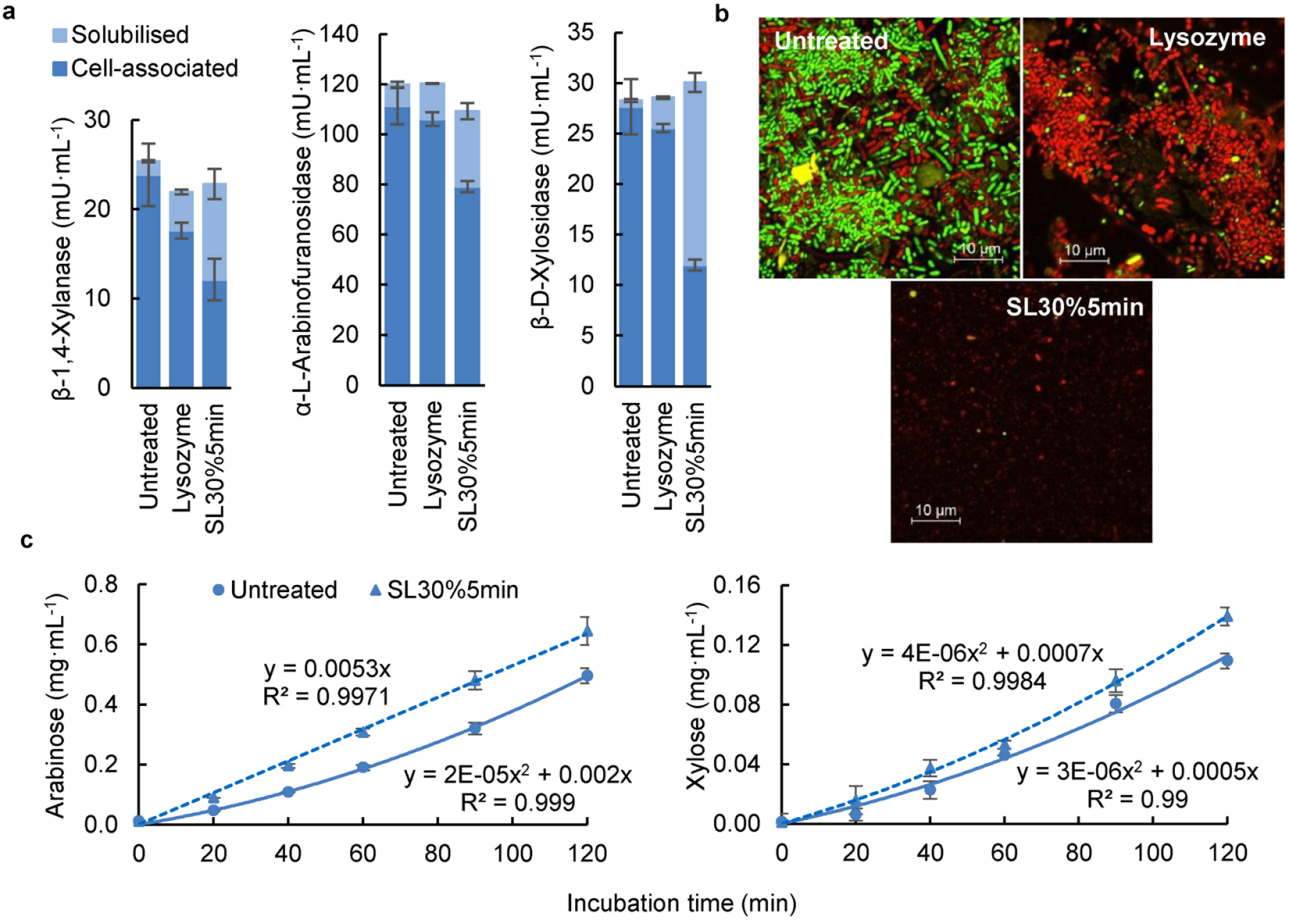
Cellular localisation of arabinoxylan-degrading enzymes. (**a**) Enzyme activities after different cell lytic treatments. Microbes were obtained from fermentation of wheat AX for 8 h. Solubilised enzymes were those in the supernatant after centrifugation (10 min, 5,000 g at 4 °C) while cell-associated enzymes were those remaining precipitated after the centrifugation. The treatment labelled ‘Lysozyme’ was performed by incubating the microbial cell suspension with lysozyme (0.5 mg/mL cell suspension) for 30 min at 37 °C with shaking (500 rpm). The treatment labelled ‘SL30%5 min’ involved a microbial cell suspension treated with sonication (5 min at 30% intensity) and followed by incubation with lysozyme (0.5 mg/mL, 30 min, 500 rpm at 37 °C). (**b**) Images from confocal laser scanning microscopy of microbes before and after lytic treatments. (**c**) Hydrolysis of 1% wheat arabinoxylan (WAX) using a bacterial cell suspension both before and after the lytic treatment.

Having shown that the microbial AX-degrading enzymes are cell-associated, we explored whether these enzymes were present on the cell surface, in the cell wall or in the cell cytoplasm. Breaking the cell walls of the microbiota is a prerequisite to release cytoplasmic enzymes. Three treatments were tested to lyse the cells: two mechanical treatments (bead-beating and sonication) and one non-mechanical treatment (enzymatic lysis). The bead-beating treatment dramatically decreased the activities of the enzyme mixture, a possible effect of enzyme proteins being absorbed onto the beads, as the concentration of enzyme proteins decreased even after bead-beating for 15 s (see Supplementary Fig. S2). In contrast, sonication for a short time (less than 10 min) did not decrease the enzyme activities (see Supplementary Fig. S2). As sonication is especially effective on Gram-negative bacterial cell walls, while lysozyme is more effective on Gram-positive bacterial cell walls, the combination of sonication and lysozyme was used to lyse the faecal microbiota post-fermentation^30^. Microbes fermented with WAX for 8 h were visualised using the live/dead stain kit to monitor microbial cell viability after the lytic treatments. As shown in Fig. 3b, before any lytic treatments, cells were almost all viable (green cells), while non-viable (red) cells and cellular debris were observed after the lytic treatments (Fig. 3b and Supplementary Fig. S3), indicating that the combined method of sonication and lysozyme effectively broke the microbes.

Following the lytic treatments, the amount of enzymes released into the solution increased; however, this was similar to the decrease in the amount of cell-associated enzymes, and the total enzyme activities were not increased (Fig. 3a and Supplementary Fig. S3). As the substrate used for β-1,4-xylanase assay was birchwood xylan dyed with Remazolbrilliant Blue R, a polymer with a molecular size of about 4 nm (see Supplementary Fig. S4), it was not expected to pass through the outer membrane of Gram-negative bacteria, or through the peptidoglycan layers of Gram-positive bacteria^31^. Therefore, the activity measured in the microbial cells before lytic treatments was likely to be from surface-located *endo*-β-1,4-xylanases. As the activity did not increase after cell lysis (Fig. 3 and Supplementary Fig. S3), this suggests that *endo*-β-1,4-xylanases produced by the microbiota are cell surface-located.

The substrates used for α-L-arabinofuranosidase and β-D-xylosidase assays were 4-nitrophenyl-α-L-arabinofuranoside (*p*NPA) and 4-nitrophenyl-β-D-xylopyranoside (*p*NPX) respectively. With a molecular weight of 271, they are expected to be able to pass through microbial cell walls^31,32^ but not through the cytoplasmic membrane, indicating that the catalytic sites of the enzymes measured before lytic treatments were located on the cell surface and/or within the cell wall. As the activity did not increase after cell lysis (Fig. 3 and Supplementary Fig. S3), this suggests that no more enzymes were released from the cytoplasm. To further explore the locations of these *exo*-enzymes, WAX was incubated with both lysed and untreated microbial cell suspensions (after 8 h fermentation). Free arabinose and xylose were released during the incubation with the untreated bacterial cell suspension, indicating the existence of surface located α-L-arabinofuranosidases and β-D-xylosidases. After treating the cell suspension by sonication (5 min at 30% intensity) and lysozyme, the concentrations of arabinose and xylose released from WAX increased, indicating that some of the cell-wall-located intracellular enzymes were released (Fig. 3c). Therefore, α-L-arabinofuranosidase and β-D-xylosidase are cell-wall associated, and present both on the bacterial surface and in the cell wall matrix. It should be noted that the different cell structures of Gram-negative and Gram-positive bacteria might also affect the locations of enzymes. The large periplasmic space in Gram-negative bacteria might be an ideal location for hydrolysis enzymes, though this is not the case for Gram-positive bacteria^33^.

The enzyme localisation studies of microbial pellets fermented with RAX for 8 h showed similar results to that of WAX (see Supplementary Fig. S5). Cell lysis of post-fermentation biomass for 4 h (WAX and RAX) also showed no increase in the total enzyme activities (see Supplementary Fig. S6). Therefore, the cellular localisation studies showed that the locations of enzymes did not change during the degradation, with all of the measurable β-1,4-xylanase found on the microbial surface, while α-L-arabinofuranosidase and β-D-xylosidase were found both on the surface and in the cell wall. The surface location of β-1,4-xylanase is consistent with a role in degrading long-chain polymers into smaller molecules before importing into the cell wall^9^. Surface-located α-L-arabinofuranosidases might also contribute to the trimming of decorated arabinoxylo-oligosaccharides before importing into the cell wall^9^. In addition, surface-located arabinofuranosidases might also work in synergy with surface-located β-1,4-xylanases, as the action of the former increases the number of un-substituted xylose units, thus producing a more preferred substrate for the latter^34^. The action of β-D-xylosidase also relies heavily on β-1,4-xylanases to produce more non-reducing terminal residues which are the main substrate for β-D-xylosidase.

### Structures of residual AX change in the medium

**Figure 4:**
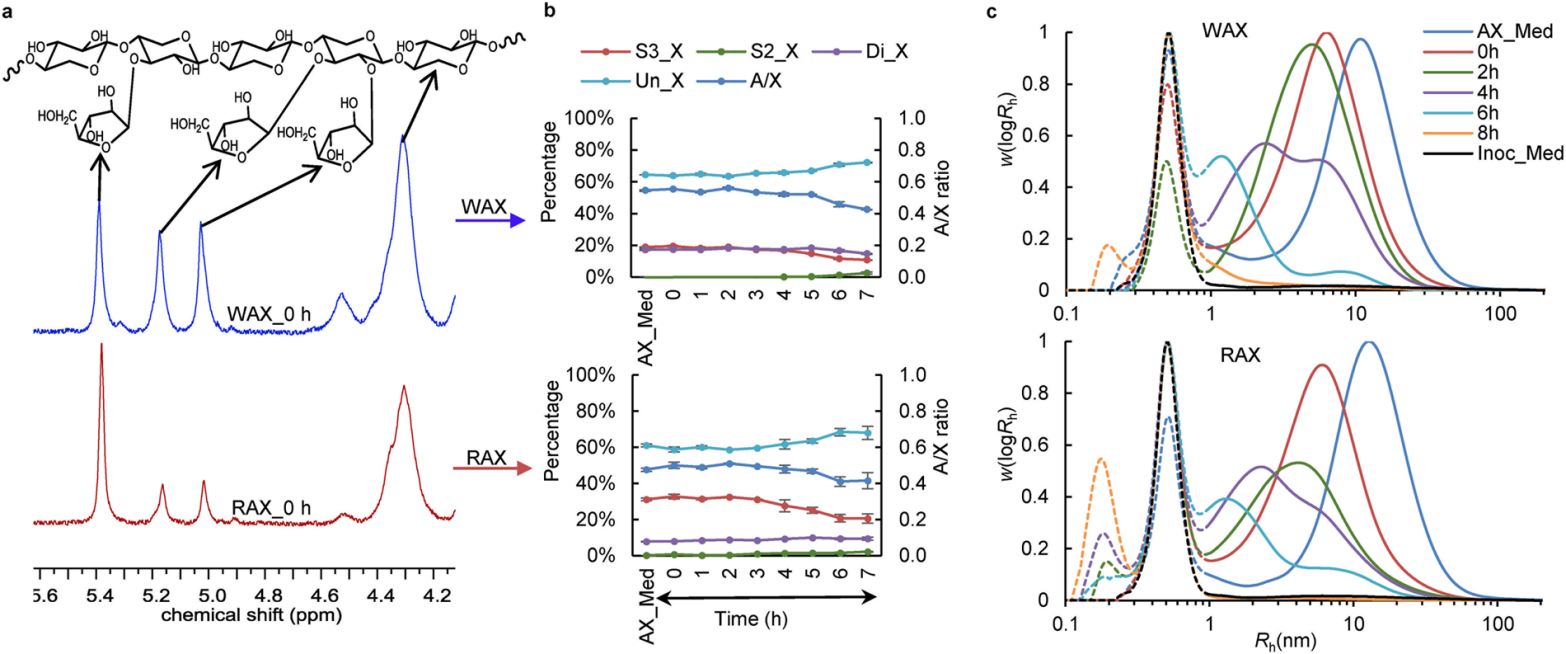
Structural analysis of residual wheat and rye arabinoxylans (AX) in the culture medium during fermentation. (**a**) ^1^H NMR spectra of wheat AX (WAX) and rye AX (RAX) at the start of fermentation. The chemical shifts of arabinose mono-substituted at C(O)3, mono-substituted at C(O)2 or di-substituted at C(O)3, and di-substituted at C(O)2 were 5.39 ppm, 5.17 ppm and 5.03 ppm respectively. The chemical shift of xylose was in the range of 4.2–4.6 ppm. The ^1^H NMR signals were assigned based on literature values^55,56^. (**b**) Substitution patterns and arabinose/xylose (A/X) ratios of residual AX. S3_X = mono-substituted xylose residues at C(O)3; S2_X = mono-substituted xylose residues at C(O)2; Di-X = di-substituted xylose residues; Un-X = un-substituted xylose residues. AX_Med was the control sample containing the substrate and the medium but without the inoculum. (**c**) Molecular size distributions (as functions of the hydrodynamic radius *R*_h_) of WAX and RAX analysed by size exclusion chromatography (SEC). Distributions normalized to the height of the maximum. Inoc_Med was the control sample containing the inoculum and the medium but without the AX substrate. Molecules in the range of 0.3 nm to 1 nm were not AX as they were present in Inoc_Med. Molecules around 0.2 nm might be small metabolites from the fermentation.

After fermentation for 5 h, substitution patterns of residual RAX and WAX (Fig. 4b) in the culture medium show decreases in the arabinose to xylose (A/X) ratio, and in the percentages of both di-substituted xylose units and mono-substituted xylose units at C(O)3. This is different to previous reports on the fermentation of insoluble or oligosaccharide forms of WAX, which showed an increase in the A/X ratio^35,36^. It is expected to be difficult for xylanases to act on heavily substituted backbone regions, and only less substituted AX regions are first cleaved by xylanase and then further degraded to monosaccharides, resulting in higher A/X ratio of residual AX^37^. This is supported by the relatively low fermentability found in previous studies^35,36^. However, water-soluble AX with medium levels of substitution are readily fermented^38^, as found in this study. Figure 4b also shows that the di-substituted xylose units decreased at a slower rate than those that are singly substituted at C(O)3, especially for RAX. This suggests that α-L-arabinofuranosidases produced by the porcine faecal microbes preferentially cleave arabinoses that are singly substituted on the xylan backbone. As the unsubstituted xyloses are more susceptible to xylanase action, this may also partly explain the higher fermentation rate of RAX than WAX in the first five hours. After 6 h, increased single arabinose substituents at C(O)2 were also observed, though at a very low percentage (3% for WAX and 2% for RAX at 7 h, Fig. 4b). The decreased single substituents at C(O)3 and the increased single substituents at C(O)2 indicate that enzymes target singly-substituted arabinoses, or arabinoses linked C(O)3 to xylose units which are di-substituted with arabinoses at C(O)2 and C(O)3. These are widely reported enzymes encoded by gut symbionts^9,39–41^.

The molecular size of the residual AX in the culture medium decreased substantially during fermentation, showing the efficient breakdown of the xylan backbone by xylanases (Fig. 4c). Unlike α-L-arabinofuranosidases, the xylanases produced had similar size-reducing actions toward WAX and RAX, despite their different substitution patterns. The size-exclusion chromatography (SEC) data also show that the large molecules of WAX and RAX had been completely degraded by 8 h, consistent with the ^1^H NMR analysis.

Given the evidence that enzymes were not secreted into the medium during active fermentation, there are two possible reasons which could account for the structural changes of residual AX in the medium: (1) there are active enzymes present in the inoculum before fermentation, and/or (2) AX is modified by cell-bound enzymes before being released back into solution.

### Enzymatically-modified AX is released from the cell surface into the medium during fermentation

**Figure 5:**
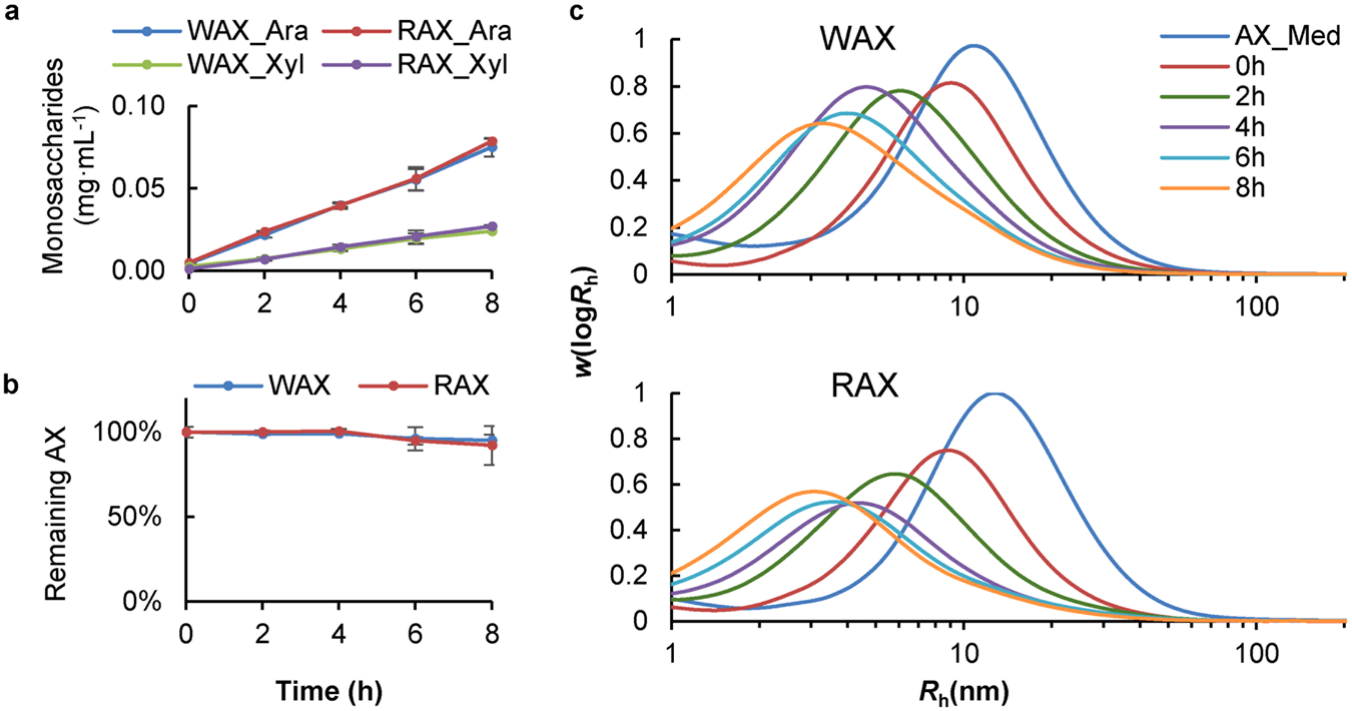
Enzymatic hydrolysis of wheat arabinoxylan (WAX) or rye arabinoxylan (RAX) with the supernatant of the faecal inoculum. (**a**) Monosaccharide concentrations in the culture medium. Ara = arabinose; Xyl = xylose. (**b**) Percentage of remaining AX in the culture medium compared with the hydrolysis at 0 h. (**c**) Molecular size distributions (as functions of the hydrodynamic radius *R*_h_) of residual AX in the culture medium. Distributions normalized to the height of the maximum and molecules smaller than 1 nm were not included as they were not AX. AX_Med was the control sample containing the substrate and the medium but without the inoculum supernatant.

By incubating WAX or RAX with the inoculum supernatant, the effects of soluble enzymes present in the inoculum on the AX structure were studied. Small amounts of monosaccharides were released, indicating activity of α-L-arabinofuranosidase and β-D-xylosidase in the inoculum supernatant. However, the amount of released monosaccharides at 8 h only accounted for around 2% of the total AX. NMR analysis showed no significant decrease in residual AX (*P* > 0.05, Fig. 5b), indicating that the decreased A/X ratio for residual AX fermented by the inoculum was not due to the small amounts of α-L-arabinofuranosidase present. This therefore shows that cell-surface located α-L-arabinofuranosidases cleave some of the arabinose substituents from the xylan backbone, with the arabinose-depleted AX being released back into solution, decreasing the residual A/X ratio in the culture medium. The released arabinose is likely to be taken up rapidly by the microbes as indicated by the very small amount of monosaccharide in the culture medium (see Supplementary Table S2).

During incubation with the inoculum supernatant, the molecular size of AX decreased, indicating β-1,4-xylanase activity in the medium, although the activity was very low (<1 mU/mL, Fig. 5c). When AX was fermented with the total inoculum, its molecular size decreased faster (Fig. 2c) than in the presence of inoculum supernatant alone with both AX species reduced to 1 nm after 8 h. This suggests that the rapid decrease in molecular size during fermentation (Fig. 2c) was not caused solely by the β-1,4-xylanase present in the inoculum solution, but was also due to cell surface located β-1,4-xylanases. We propose that the long chain AX was degraded by the surface located β-1,4-xylanases into smaller oligosaccharides, with some of the resulting lower molecular size AX being released back into the solution.

### Competitive and co-operative utilisation of AX by the porcine faecal inoculum

**Figure 6:**
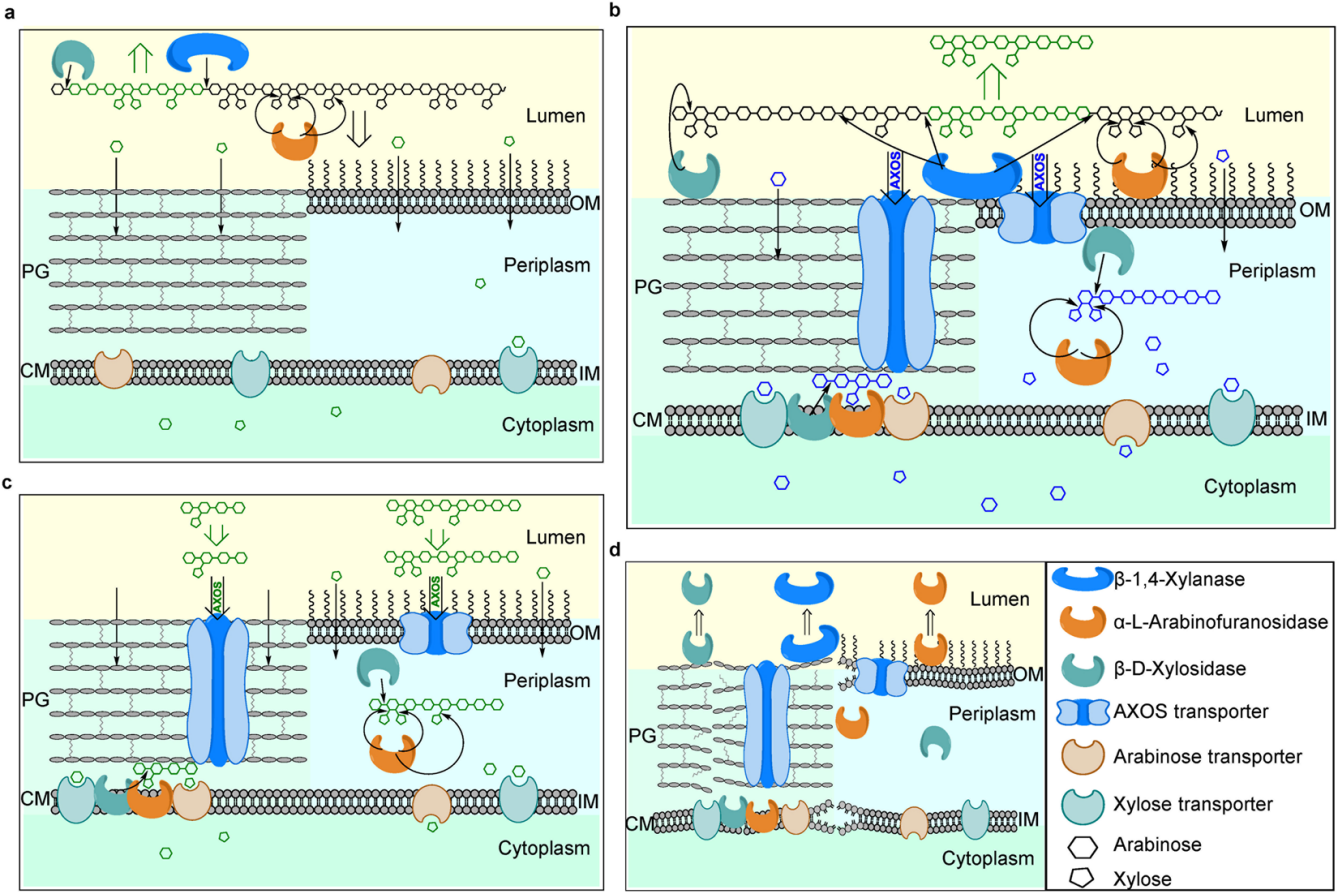
A model for the utilisation of arabinoxylan (AX) by colonic microbiota. The left part of each panel shows a schematic structure of gram-positive bacterial cell wall and the right shows that of gram-negative bacteria. Gram-positive and negative bacteria share the same cytoplasmic/inner membrane features. PG: peptidoglycan; CM: cytoplasmic membrane; OM: outer membrane; IM: inner membrane. (**a**) Degradation of AX by soluble enzymes in the gut lumen. (**b**) Degradation of AX by a suite of surface located enzymes to hydrolyse AX before importing into the cell wall or releasing back into the gut lumen. (**c**) Degradation of arabinoxylo-oligosaccharides (AXOS): bacteria lacking surface-located enzymes may harbour cell-wall-located intracellular enzymes, which are unable to utilise AX but are capable of utilising AXOS. (**d**) Release of AX-degrading enzymes during biomass turnover.

To summarize the findings of this study, a model was developed to describe the mechanisms by which porcine faecal bacteria utilised AX (Fig. 6). The model does not include any specific surface binding proteins that might contribute to the recognition and acquisition of AX or AXOS, except for the AXOS transporter in the outer membrane of Gram-negative bacteria or peptidoglycan layers of Gram-positive bacteria^12^, and the monosaccharide transporters in the cytoplasmic membrane. We show here that the large molecules of AX may be partially degraded by soluble enzymes present in the inoculum supernatant (which can be thought of as representing the gut lumen) (Fig. 6a). The resulting monosaccharides are rapidly utilised, while the oligosaccharides are further degraded by the microbes. AX-degrading bacteria produce a suite of surface-located enzymes to hydrolyse AX before importation into the cell wall (Fig. 6b). Of the lower molecular size and arabinose-depleted AX and AXOS molecules produced during fermentation, some are taken up by the bacteria and the rest are released back into the gut lumen with potential to be utilised by other microbes. AXOS molecules that are taken up by the bacteria are further degraded into monosaccharides by cell-wall-located intracellular α-L-arabinofuranosidases and β-D-xylosidases, which also include enzymes associated with the cytoplasmic membrane.

AXOS released back into the gut lumen may support the growth of AXOS-degrading bacteria (Fig. 6c), which are incapable of utilizing the large polymers because they lack the surface-located enzymes^15,42^. While the release of monosaccharides into the culture medium from Bifidobacteria growing on AXOS and xylo-oligosaccharides (XOS) has been reported, the results were based on endpoint determinations, so this may be due to the release of enzymes during microbial autolysis^43^. The preference for consuming short XOS over xylose by some Bifidobacteria is consistent with the intracellular hydrolysis of these oligosaccharides^29,43–46^. However, in our study, as no measurable enzymes were found in the cytoplasm, the (A)XOS taken up by the bacteria are probably degraded into their component sugars by cell-wall-located α-L-arabinofuranosidases and β-D-xylosidases. These bacteria might encode relatively fewer degrading enzymes compared to AX-degrading bacteria, but tend to upregulate the syntheses of saccharide transporters^47^.

During biomass turnover, the lysed bacteria release some of the AX-degrading enzymes into the gut lumen (Fig. 6d), and these released enzymes are still active against AX present in the gut lumen. Recent studies of the degradation of xylan by *B. ovatus* showed xylanase activity in the culture medium, and it was hypothesised that xylanases are secreted into the medium as “public goods”^15^. Here we show that, during the active fermentation of AX, AX-degrading enzymes are not secreted into the culture medium, and only the lower molecular size and arabinose-depleted AX molecules produced during the fermentation are “public goods”. During active biomass turnover, however, these AX-degrading enzymes are released into the culture medium and subsequently become “public goods”.

Previous studies based on bioinformatics analyses have suggested that xylan-degrading enzymes of Gram-negative bacteria are cell-associated^9^. However, the largest number of colonic bacteria belong to the phylum Firmicutes^8^, many of whose species have a Gram-positive cell wall structure. Therefore, previous studies on enzyme locations have not used systems representative of the diverse population of the colonic bacterial community. We believe that the present work is the first time that enzyme assays and polysaccharide analysis have provided strong evidence for the locations of AX-degrading enzymes encoded by (porcine) faecal microbiota. The locations of AX-degrading enzymes enable us to predict how AX is degraded by the microbiota. In one mechanism (selfish or competitive), the cell wall-associated enzymes are not public goods for the other bacteria during the degradation, and thus enable the competitive utilisation of AXs by the enzyme-producing bacteria. Previous studies have shown that an individual bacterial species can harbor the genes necessary for synthesizing all the enzymes needed to degrade complex polysaccharides^1,9,14^, which indicates that they have evolved an efficient polysaccharide-degrading system within a competitive environment. This study has also demonstrated two types of mechanisms that lead to co-operative rather than selfish/competitive utilisation of AX. During biomass turnover, some of the enzymes are released into the medium (gut lumen) and become available for degrading AX into AXOS, arabinose and xylose which may then be taken up by species which lack one or more of the package of enzymes present in “selfish” bacteria. A second type of co-operative mechanism involves the release of partially hydrolysed AX following the action of cell-surface-associated enzymes. The data presented show that all types of enzyme digested products can be released – lower molecular size AX, arabinose-depleted AX, arabinose and xylose. All of these products are therefore available for utilisation by members of the bacterial community which lack one or more of the enzyme activities required for AX hydrolysis.

In conclusion, this study has provided a model for the utilization of AX by the porcine faecal microbiota: a competitive/selfish and co-operative utilisation. In the colonic environment, where various glycans and related breakdown products are valuable nutrients, it is likely that some bacteria have developed a sophisticated “selfish” degrading system, including the capture, binding and hydrolysis of glycans, and transportation of the degraded products, like some Bacteroidetes^1,9,14^. However, if this were the dominant mode of utilisation of dietary fibre polysaccharides, a limited number of species would be expected to out-compete others, leading to a low microbial diversity. This is at odds with the principle that a diverse microbiome is associated with lower risks of non-communicable diseases^48^ and the overwhelming evidence that a diet rich in dietary fibre is protective against the same diseases. We therefore propose that the two co-operative or “public goods” mechanisms identified here are critical for providing microbiome diversity, consistent with the health-benefiting properties of dietary fibre polysaccharides^49^. In the future, it would be necessary to study which bacteria initiate the degradation of AX, and which bacteria take advantage of the released oligosaccharides. The colonisation of specific microbes, together with the consumption of specific glycans, might make it possible to characterize the essential elements of a diverse and healthy microbiota, with the ultimate health benefits that this could confer.

## Methods

### *In vitro* fermentation of AX with a porcine faecal inoculum

*In vitro* fermentation of wheat AX (Megazyme, product code: P-WAXYM) and rye AX (Megazyme, product code: P-RAXY) was undertaken according to the method described by Williams *et al*.^50^. A typical fermentation bottle contained approximately 0.4 g of substrate and 82 mL of the anaerobic, sterile, semi-defined culture medium^51^. Faeces from pigs fed a controlled diet for ten days was used as inoculum. All methods were carried out in accordance with relevant guidelines and regulations. All animal handling procedures and related experimental protocols were approved by the University of Queensland Animal Ethics Committee (SAFS/111/13/ARC). The diet (see Supplementary Table S1), based on highly digestible maize starch and fishmeal, was formulated to be as free as possible of AX to avoid adaptation of the microbiota to the tested AX substrates. The faeces were diluted 6 times with pre-warmed (39 °C) saline solution and filtered through four layers of muslin cloth. Fermentation commenced when 5 mL of the above prepared faecal mixture was inoculated into each bottle, which were incubated at 39 °C and the fermentation proceeded to 48 h. Two blanks were included. AX_Med contained the substrate (WAX or RAX) and the medium but no inoculum, for which two samples were taken for each substrate, one at 0 h and the other at 48 h. Ino_Med contained the inoculum and the medium, but no substrate, for which one sample was taken at each timed removal period. At specific time points, the serum bottles were removed from the incubator, and plunged into an ice-water bath for 20 minutes to retard the microbial fermentation activity.

Post-fermentation, 3 mL of the top liquid phase (avoiding particulate matter) was taken from individual sample bottles for SCFA analysis and another 3 mL for NH_3_ analysis, then the rest of the contents were split across two 50 mL tubes for centrifugation (10 min, 5,000 g at 4 °C) and separation into supernatant (culture medium) and pellet fractions. Various aliquots of the supernatant (ranging from 1 mL to 40 mL) were collected for analyses of enzymes, monosaccharides and molecular structures (after freeze-drying). The pellets were separated into aliquots of 1 mL, snap-frozen and stored at –80 °C for subsequent analyses of microbial dry matter (after freeze-drying) and various enzyme activities.

### Dry matter

Post-fermentation pellet aliquots were thawed (4 °C) and centrifuged (10 min, 5,000 g at 4 °C). Excess supernatant was removed and the pellets were freeze-dried and weighed. In order to avoid washing away any of the AX or AXOS attached to the pellets, dry matter was determined without washing the pellets. However, as ^1^H NMR analysis showed no detectable residual AX on the pellets, it appeared that the pellets were mainly microbes. Data are expressed by the dry weight of pellet per gram dry matter of WAX or RAX used.

Dry matter of the substrates (WAX and RAX) was determined through oven drying at 105 °C.

### Whole cell assays

Post-fermentation pellet aliquots were thawed (4 °C), centrifuged (10 min, 5,000 g at 4 °C), washed, and resuspended in PIPES buffer (50 mM, pH 6.8). Enzyme activities were evaluated using colorimetric methods (described below).

### Cellular localisation studies

Microbial pellets collected 8 h after WAX fermentation were used for cellular localisation studies. The pellets were thawed at 4 °C, centrifuged (10 min, 5,000 g at 4 °C), washed and resuspended in sufficient PIPES buffer (50 mM, pH 6.8) to make it the same concentration as at the end of the fermentation. The cell suspension was treated by sonication under various conditions (from 1 to 5 min at 30% to 40%) and then followed by enzymatic treatment. Sonication was undertaken by using an ultrasonic processor (Vibra-Cell^TM^, VCX 750) and a microprobe (3 mm in diameter). Enzymatic treatment was performed by incubating the microbial cell suspension with lysozyme (0.5 mg lysozyme per mL cell suspension, from chicken egg white, L6876 Sigma) for 30 min at 37 °C with shaking (500 rpm). Cell viability was monitored using the live/dead bacterial viability kit (L7012, BacLight ^TM^, Thermo Fisher Scientific, USA) and a CLSM (CZ Microscopy GmbH 07745 Jena, Germany), where cells with intact membranes are stained green with SYTO 9, while those with damaged membranes are stained red with propidium iodide. After the lytic treatments, the cell suspension was centrifuged (10 min, 5,000 g at 4 °C), and the enzymes measured in the supernatant were classified as solubilised enzymes. The pellets which were resuspended in PIPES buffer and enzyme activities then measured, were referred to as cell-associated enzymes. Cell suspension without any lytic treatment was used as a control. Enzyme activities were assayed using colorimetric methods (described below). The activities of α-L-arabinofuranosidase and β-D-xylosidase of cells without treatment and those treated with sonication (5 min at 30% intensity) and lysozyme (0.5 mg/mL) were also assayed with 1% WAX (described below).

### Enzymatic hydrolysis of AX with the inoculum supernatant

The inoculum was centrifuged (20 min, 14,000 g at 4 °C) and the supernatant was collected. Enzymatic hydrolysis of WAX and RAX with the inoculum supernatant was conducted under the same condition as the fermentation but at a smaller scale, which contained approximately 200 mg of the substrate, 41 mL of the culture medium and 2.5 mL of the inoculum supernatant. WAX and RAX were hydrolysed for up to 8 h.

### ^1^H NMR and SEC analysis

Before ^1^H NMR and SEC analyses, the samples were freeze-dried. ^1^H NMR measurements were performed at 298 K on a Bruker 500 MHz spectrometer. DMSO-*d*_6_ was used as the solvent and trimethylsilyl propanoic acid sodium salt (TSP) in D_2_O was added as an internal reference^52^. Trifluoroacetic acid (TFA) was used to shift the water peak away from the diagnostic anomeric proton signals^53^.

For SEC analysis, the freeze-dried supernatant was washed with 90% ethanol and then centrifuged to remove the soluble lipids and proteins. The collected pellets were air-dried and then dissolved in DMSO containing 0.5% LiBr. The prepared samples were analysed by SEC according to the method described by Wang *et al*.^54^.

### Glycoside hydrolase assays

Colorimetric methods: The activity of β-D-1,4-xylanase was measured by incubating the enzyme solution (0.1 mL) with azo-xylan (0.1 mL, Megazyme, product code: S-AXBL) for 1 h at 39 °C with shaking (500 rpm). The reaction was terminated by the addition of 95% ethanol (0.5 mL). Non-hydrolysed substrate of high molecular weight was precipitated while the breakdown products of low molecular weight remained in solution. The absorbance of the supernatant after centrifugation was measured at 595 nm with a plate reader (BMG Labtech, Germany). *Endo*-1,4-β-xylanase M6 (2–50 mU/mL, rumen microorganism, Megazyme, product code: E-XYRU6) was used as a standard. The activities of β-D-xylosidase and α-L-arabinofuranosidase were evaluated by incubating the enzyme solution (0.1 mL) with 0.1 mL of 5 mM 4-nitrophenyl-β-D-xylopyranoside (Sigma-aldrich, N2132) and 4-nitrophenyl-α-L-arabinofuranoside (Sigma-aldrich, N3641) respectively. The reaction was ended by the addition of tris(hydroxymethyl)aminomethane (2% in water, w/v). The 4-nitrophenol produced was determined colorimetrically at 405 nm using a plate reader, with 4-nitrophenol (Sigma-Aldrich, 73560) as the standard (0.05–0.6 mM). Enzymatic activities were expressed in units (U), one unit of β-xylosidase/α-L-arabinofuranosidase activity being defined as the amount of enzyme required to release one µmol of 4-nitrophenol per min under the above conditions.

WAX/RAX substrates: The activities of β-D-xylosidase and α-L-arabinofuranosidase were also evaluated by incubating the enzyme solution with WAX/RAX solution (1% in water, w/v). The cell suspensions (with or without the lytic treatment) were diluted five times with PIPES buffer (50 mM, pH 6.8) and incubated with equal volumes of WAX/RAX solution for up to 120 min at 39 °C with shaking (500 rpm). The reaction was stopped by heating in boiling water for 20 min. The concentrations of released arabinose and xylose were measured using arabinose (Megazyme, product code: K-ARGA) and xylose (Megazyme, product code: K-XYLOSE) assay kits.

All the enzyme assays were performed in duplicate under aerobic conditions.

### Experimental group size and statistical analysis of data

At least two technical replicates were performed for all quantitative assays. The fermentation of WAX and RAX was performed as two biological replicates while the enzymatic hydrolysis of WAX and RAX using the inoculum supernatant was performed as three replicates. The error bars in figures represent standard deviations of the mean from biological replicates. SEC data are the means of biological replicates.

All parameters were tested for significant differences (effects of AX and removal time, and their interaction) using the Tukey-Kramer multiple comparison procedure as defined by:

1 $${\rm{y}}=\mu +{\rm{A}}{\rm{X}}{i}+{\rm{R}}{\rm{T}}{j}+({\rm{A}}{\rm{X}}{i}\times {\rm{R}}{\rm{T}}{j})+\varepsilon {i}{j}$$

Here y is the dependent variable, μ is the mean, AX_i_ is the effect of AX, RT_*j*_ is the effect of removal time, AX_i_ ×RT_*j*_ is the interaction between AX and removal time, and ε_*i*__*j*_ is the error term. Statistical analyses were performed using SAS Proc GLM (significant difference) procedures (SAS, SAS 9.4 for Windows, SAS Institute Inc, Cary, NC, USA, 9.4 Edn. 2013).

### Data availability

All data generated or analysed during this study are included in this published article (and its Supplementary Information files). The datasets generated during and/or analyzed during the current study are available from the corresponding author on reasonable request.

## Electronic supplementary material

Supplementary Information
