# Supplementary material for: Mechanisms of utilisation of arabinoxylans by a porcine faecal inoculum: competition and co-operation
Source: Sci Rep. 2018 Mar 14;8:4546. doi: 10.1038/s41598-018-22818-4 (PMC5852058; doi:10.1038/s41598-018-22818-4)
Supplement: Supplementary file 1 — Supplementary Information [file 41598_2018_22818_MOESM1_ESM.pdf]

Supplementary Information

Title: Mechanisms of utilisation of arabinoxylans by a porcine faecal inoculum: competition and co-operation

Guangli Feng[1], Bernadine M. Flanagan[1], Deirdre Mikkelsen[1], Barbara A. Williams[1], Wenwen Yu[2], Robert G. Gilbert[2,3], & Michael J. Gidley[1,*]

[1] ARC Centre of Excellence in Plant Cell Walls, Centre for Nutrition and Food Sciences, Queensland Alliance for Agriculture and Food Innovation, The University of Queensland, St Lucia, QLD, Australia 4072.

[2] Centre for Nutrition and Food Sciences, Queensland Alliance for Agriculture and Food Innovation, The University of Queensland, St Lucia, QLD, Australia 4072.

[3] Joint International Research Laboratory of Agriculture and Agri-Product Safety, College of Agriculture, Yangzhou University, Yangzhou, Jiangsu Province 225009, China

*Correspondence: m.gidley@uq.edu.au

Supplementary methods

Effects of mechanical treatment on enzyme activities

An enzyme mixture containing 100 mU/mL of α-L-arabinofuranosidase (*Aspergillus niger*, Megazyme, product code: E-AFASE), 100 mU/mL of β-D-xylosidase (*Selenomonas ruminantium*, Megazyme, product code: E-BXSR) and 200 mU/mL of β-1,4-xylanase (rumen microorganism, Megazyme, product code: E-XYRU6) in sodium acetate buffer (pH 5.0, 0.1 M) with 0.5 mg/mL bovine serum albumin (BSA) was prepared. Aliquots of 0.7 mL of the enzyme mixture were added into O-ring tubes pre-filled with 1.0 g silicon beads (0.1 mm in diameter). Bead beating was carried out with a Tissuelyser II (Qiagen®) at various conditions (30-180 s at 20-30 Hz). Aliquots of 3 mL of the above enzyme mixture were treated with an ultrasonic processor (Vibra-Cell™, VCX 750) at 40% of its maximum intensity, and a microprobe with a diameter of 3 mm was used. The remaining enzyme activities after the treatments were assayed using colorimetric methods.

Test of absorptive effect of enzymes onto silicon beads

An enzyme mixture with a total protein concentration of 21.23 µg/mL in PIPES buffer (50 mM, pH 6.8) was prepared, containing 15.62 µg/mL of α-L-arabinofuranosidase (*Aspergillus niger*), 4.23 µg/mL of β-D-xylosidase (*Selenomonas ruminantium*) and 1.38 µg/mL of β-1,4-xylanase (rumen microorganism). The enzyme mixture was treated with bead beating at various conditions (15-60 s at 20-30 Hz), and enzyme concentrations in the solution before/after bead beating were measured with Micro BCA™ protein assay kit (catalogue: 23235, ThermoFisher Scientific).

Cellular localisation studies

Microbial pellets fermented with rye arabinoxylan (RAX) for 8 h were treated with sonication for 5 min at 30% intensity, followed by treatment with lysozyme (0.5 mg/mL, 30

min, 37 °C and 500 rpm, lysozyme was from chicken egg white, L6876 Sigma). Enzyme activities were assayed without centrifugation using both colorimetric methods and RAX solution (RAX, 1%, w/v). Microbial cell suspension without any lytic treatments was used as control.

Microbial pellets fermented with either wheat AX (WAX) or RAX for 4 h were treated with sonication for 5 min at 30% intensity, followed by the treatment of lysozyme (0.5 mg/mL, 30 min, 37 °C and 500 rpm). Enzyme activities were assayed without centrifugation using the colorimetric methods. Microbial cell suspension without any lytic treatments was used as control.

Analyses

Concentrations of arabinose and xylose were measured with L-arabinose assay kit (Megazyme, product code: K-ARGA) and D-xylose assay kit (Megazyme, product code: K-XYLOSE), respectively.

Short chain fatty acid (SCFA) and ammonia were analysed using methods described by Williams *et al*[1].

Supplementary Tables

Supplementary Table S1 Composition of the defined pig diet

Ingredients	Defined Diet (g/kg)
Maize starch	504.8
Fishmeal	200.0
Soya isolate	50.0
Dextrose	150.0
Soya oil	15.0
Cellulose (Arbocel)	50.0
Premix (vitamin, mineral)	10.0
Limestone	2.5
Monocalcium phosphate	1.5
$KHCO_3$	12.0
L-lysine HCl	0.6
DL-methionine	2.0
L-threonine	1.0
L-tryptophan	0.6

Supplementary Table S2 Monosaccharide concentrations in the culture medium during fermentation of wheat arabinoxylan (WAX) or rye arabinoxylan (RAX) with a porcine faecal inoculum.

Time (h)	WAX ($mg \cdot mL^{-1}$)		RAX($mg \cdot mL^{-1}$)	
	Arabinose	Xylose	Arabinose	Xylose
0	≤0.01	≤0.01	≤0.01	≤0.01
1	≤0.01	≤0.01	≤0.01	≤0.01
2	≤0.01	≤0.01	≤0.01	≤0.01
3	≤0.01	≤0.01	≤0.01	≤0.01
4	≤0.01	≤0.01	≤0.01	≤0.01
5	0.02±0.000	≤0.01	0.02±0.003	≤0.01
6	0.04±0.012	≤0.01	0.03±0.005	≤0.01
7	0.04±0.000	0.02±0.000	0.03±0.003	≤0.01
8	0.03±0.000	0.02±0.003	≤0.01	≤0.01
10	≤0.01	≤0.01	≤0.01	≤0.01
12	≤0.01	≤0.01	≤0.01	≤0.01
15	≤0.01	≤0.01	≤0.01	≤0.01
18	≤0.01	≤0.01	≤0.01	≤0.01
24	≤0.01	≤0.01	≤0.01	≤0.01
48	≤0.01	≤0.01	≤0.01	≤0.01

Supplementary Figures

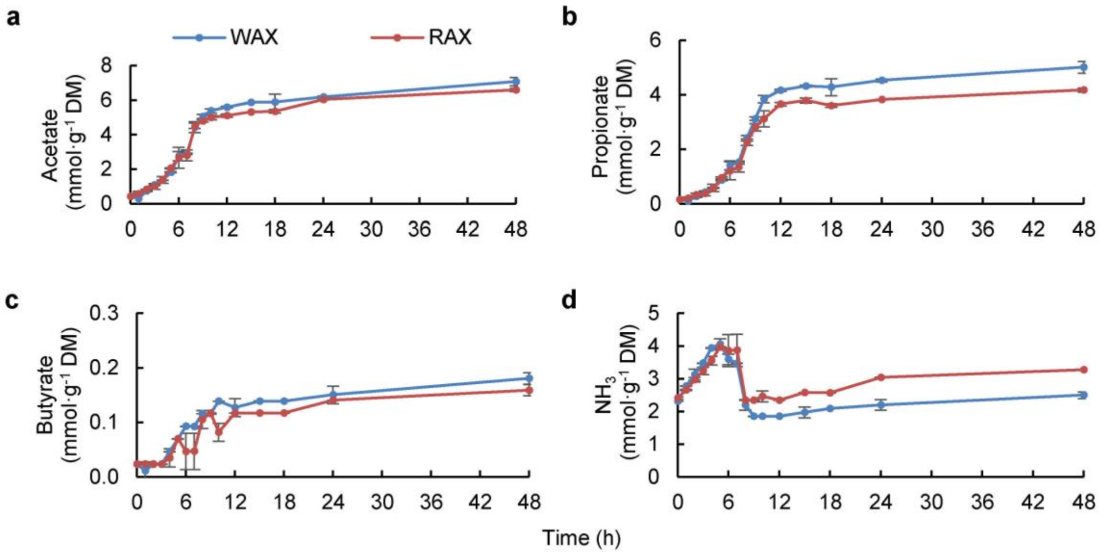

Supplementary Figure S1 Short chain fatty acids (**a**: acetate; **b**: propionate; **c**: butyrate) and NH_3 (**d**) produced by the fermentation of wheat or rye arabinoxylan with a porcine faecal inoculum.

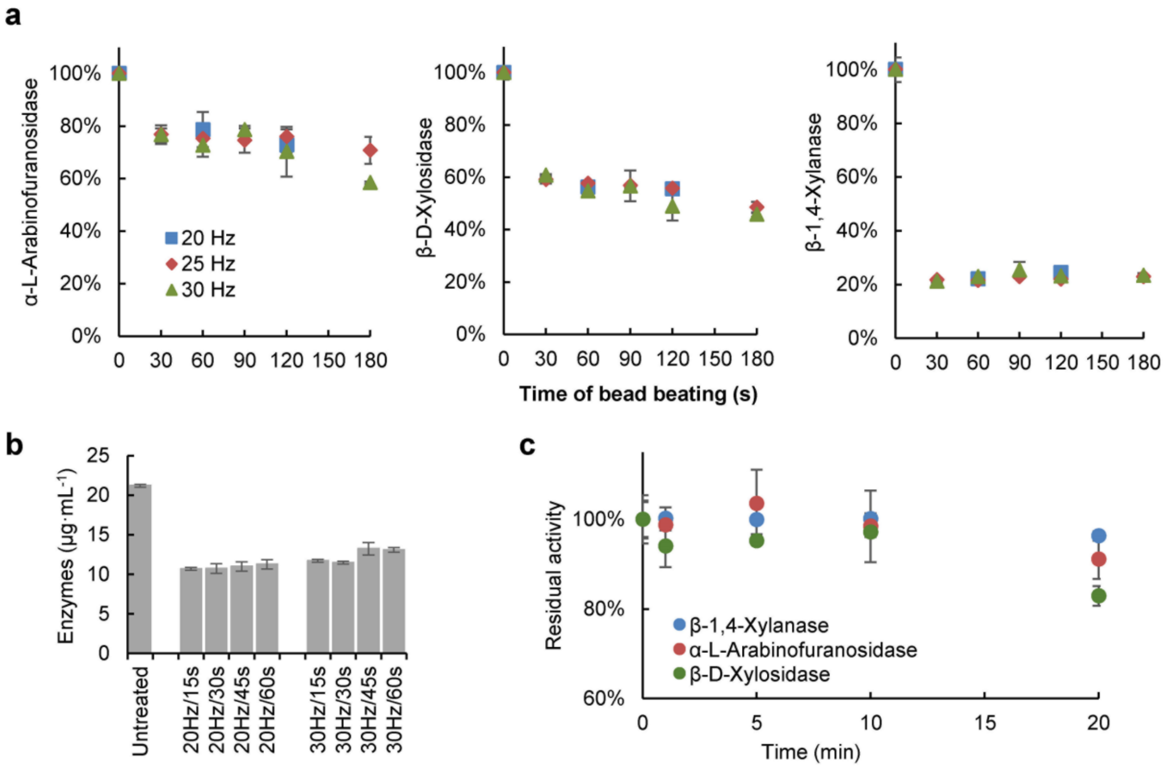

Supplementary Figure S2 Effects of mechanical treatment on enzyme activities. Effects of bead beating (**a**) and sonication (**c**) on enzyme activities. (**b**) Absorptive effect of enzymes onto silicon beads during bead beating.

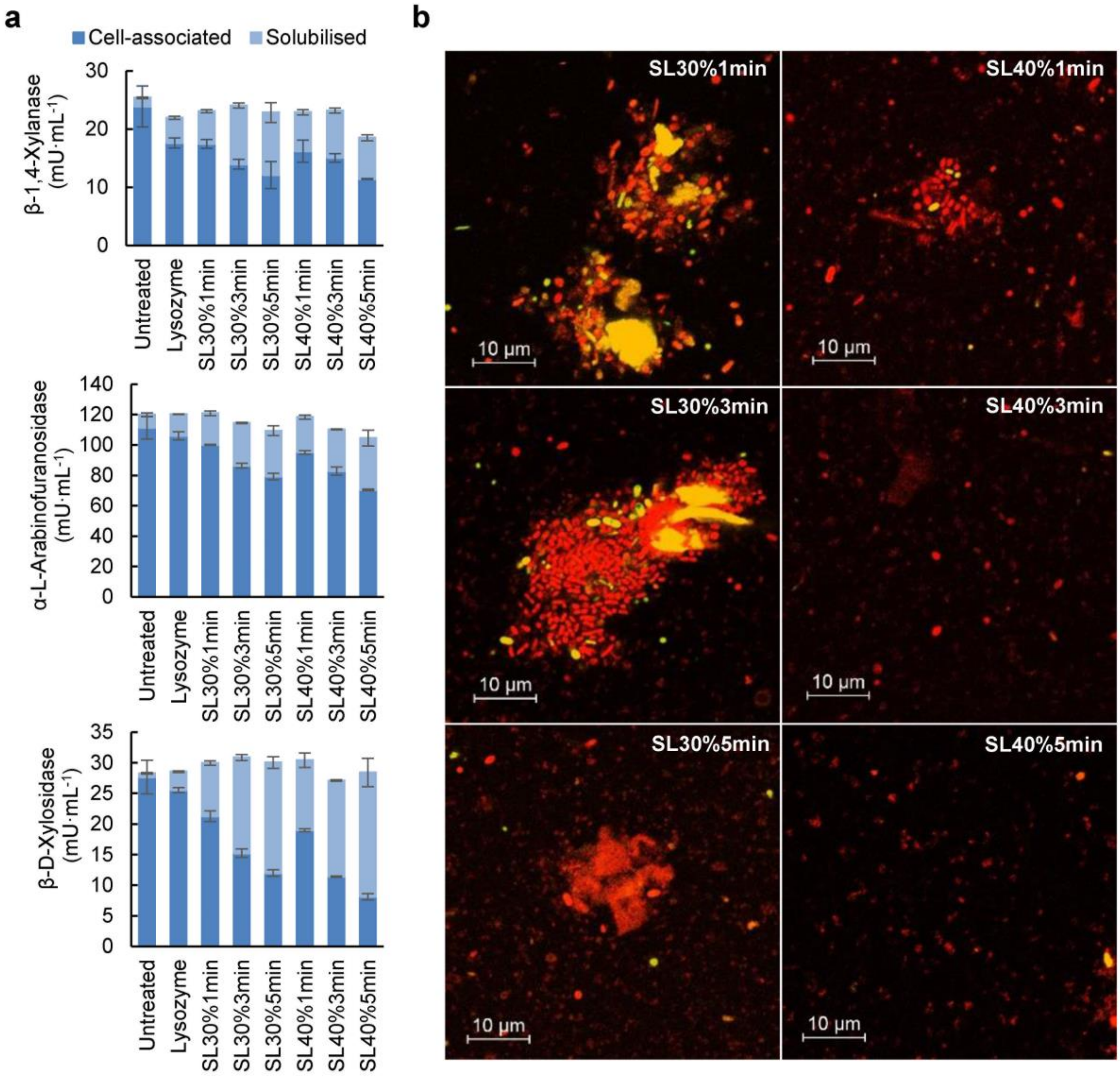

Supplementary Figure S3 Cell lysis of microbes obtained by fermentation of wheat AX for 8 h with a faecal inoculum. (**a**) Enzyme activities (β-1,4-xylanase, α-L-arabinofuranosidase and β-D-xylosidase) before and after different cell lysis treatments. Solubilised enzymes are enzymes in the supernatant after centrifugation (10 min at 5,000 g and 4 °C) while cell-associated enzymes are enzymes remaining attached to the microbial pellets and precipitated after the centrifugation. Lysozyme treatment was undertaken by incubating the microbial cell suspension with lysozyme (0.5 mg/mL) for 30 min at 37 °C and 500 rpm. The treatment labelled SL involved a microbial cell suspension treated with sonication (1-5 min at 30-40% intensity) followed by incubation with lysozyme (0.5 mg/mL, 30 min, 500 rpm at 37 °C). A microprobe of 3 mm in diameter was used for sonication. (**b**) Images from confocal laser scanning microscopy of microbes post lytic treatments.

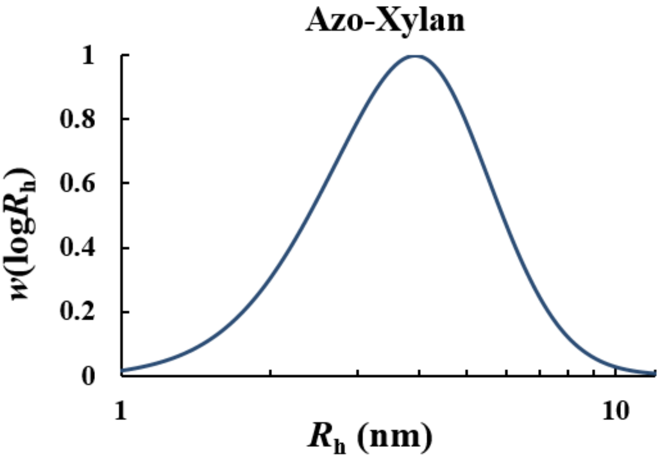

Supplementary Figure S4 Molecular size (as functions of the hydrodynamic radius R_h) of azo-xylan by using size exclusion chromatography (SEC). Distributions normalized to the height of the maximum.

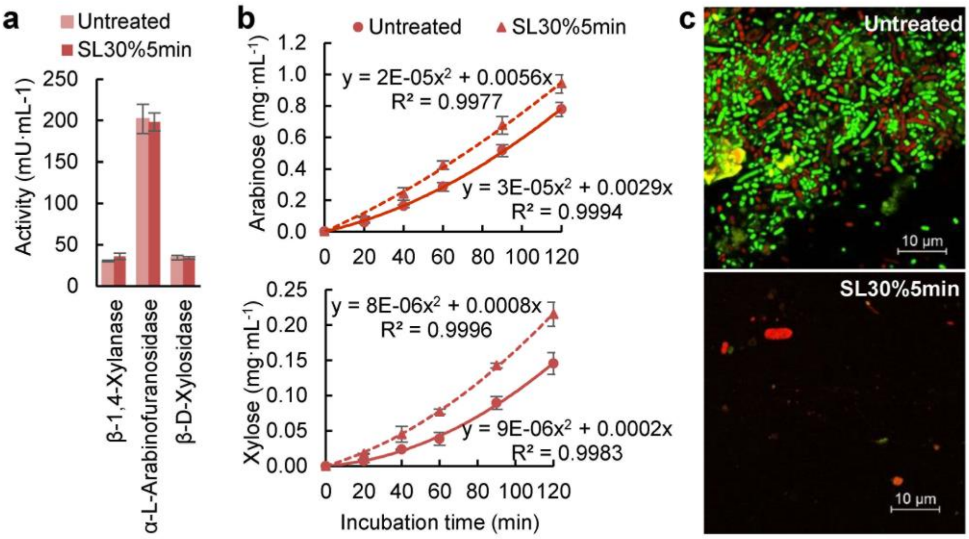

Supplementary Figure S5 Cell lysis of microbes obtained by fermentation of rye AX (RAX) for 8 h with a porcine faecal inoculum. (**a**) Enzyme activities (β-1,4-xylanase, α-L-arabinofuranosidase and β-D-xylosidase) before and after cell lysis treatment. The treatment labelled SL30%5min involved a microbial cell suspension treated with sonication (5 min at 30% intensity) and followed by incubation with lysozyme (0.5 mg/mL, 30 min, 500 rpm at 37 °C). (**b**) Hydrolysis of rye arabinoxylan (RAX) with microbial cell suspension before and after the lytic treatment. RAX of 1% in deionised water was used. (**c**) Images from confocal laser scanning microscopy of microbes before and after the lytic treatment.

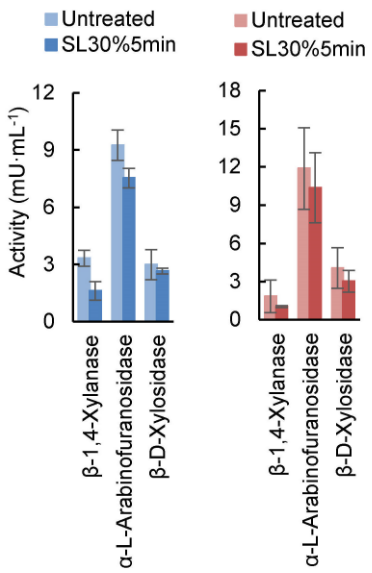

Supplementary Figure S6 Cell lyses of microbes obtained by fermentation of wheat or rye AX (WAX or RAX) for 4 h with a porcine faecal inoculum. The treatment labelled SL30%5min involved a microbial cell suspension treated with sonication (5 min at 30% intensity) followed by incubation with lysozyme (0.5 mg/mL, 30 min, 500 rpm at 37 °C).

Supplementary Reference

1 Williams, B. A. *et al*. In vitro fermentation kinetics and end-products of cereal arabinoxylans and (1, 3; 1, 4)-β-glucans by porcine faeces. *Journal of Cereal Science* **53**, 53-58 (2011).
